# *Nigella sativa *and its main constituent, thymoquinone protect against glycerol-induced acute kidney injury in rats

**DOI:** 10.22038/AJP.2022.20921

**Published:** 2022

**Authors:** Elham Naderi, Abolfazl Khajavi Rad, Somayeh Nazari, Majid Khazaei, Samira Shahraki, Sara Hosseinian

**Affiliations:** 1 *Department of Physiology, School of Medicine, Mashhad University of Medical Sciences, Mashhad, Iran*; 2 *Applied Biomedical Research Center, Mashhad University of Medical Sciences, Mashhad, Iran*; 3 *Medicinal & Natural Products Chemistry Research Center, Shiraz University of Medical Sciences, Shiraz, Iran*; 4 *Department of Physiology, School of Medicine, Zahedan University of Medical Sciences, Zahedan, Iran*

**Keywords:** Nigella sativa, Thymoquinone, Rhabdomyolysis

## Abstract

**Objective:**

Rhabdomyolysis is a life-threatening disease caused by releasing myoglobin from injured myocytes, which results in acute kidney injury. In this study, the effect of aqueous-alcoholic extract of *Nigella sativa* (NS) and thymoquinone (TQ) on rhabdomyolysis-induced kidney damage in rats was investigated.

**Materials and Methods:**

There were five groups of rats (n=8): Control, rhabdomyolysis, rhabdomyolysis treated with NS aqueous-alcoholic extract (200 and 400 mg/kg) and TQ (15 mg/kg). Treatments were given for 7 days (two days before and four days after glycerol injection). Glycerol was injected intramuscularly on the third day of the experiment for induction of rhabdomyolysis. Renal function parameters on the first, fourth, and seventh days of the experiment and renal oxidative stress and histological changes at the end of this study were assessed.

**Results:**

Glycerol injection caused a significant increase in serum level of urea, creatinine, creatine phosphokinase, urine output and tissue MDA compared to the control animals (p<0.05-0.001). Administration of NS extract and TQ significantly decreased serum urea and creatinine on days 4 and 7, creatine phosphokinase on day 4, and urine output on day 7 compared to the rhabdomyolysis group (p<0.05-0.001). Compared to the rhabdomyolysis group, treatment with NS extract and TQ improved kidney histological abnormalities (p<0.01-0.001). The catalase enzyme activity in the group treated with NS 400 mg/kg and thiol content in the NS 400 mg/kg and TQ groups were significantly higher than those of the rhabdomyolysis group (p<0.05-0.01).

**Conclusion:**

*NS* extract and to some extent TQ protect the kidney from rhabdomyolysis-induced injury.

## Introduction

Rhabdomyolysis (RM) is skeletal muscle destruction and release of the contents of skeletal muscle cells into the plasma. Some of these cellular contents, especially myoglobin, can be thoroughly filtered from the glomeruli and lead to acute kidney injury (AKI) (Panizo et al., 2015 ▶). Rhabdomyolysis is caused by various causes such as drugs, trauma, metabolic disorders, malignant hyperthermia, overheating, electrolyte changes, diabetic ketoacidosis, non-diabetic hyperosmolar coma, hyperthyroidism or hypothyroidism, bacterial and viral infections, genetic diseases, connective tissue diseases, muscle ischemia, and narcotics, of which trauma is the main cause (Khan, 2009 ▶; Parekh et al., 2012 ▶). The main pathophysiological features of RM are decreased ATP and the following intracytosolic calcium increase (Coco and Klasner, 2004 ▶; Zager, 1996 ▶). Decreased cell energy due to ATP depletion causes dysfunction of the Na^+^-K^+^ ATPase and the Ca^2+^-ATPase pumps, leading to increased intracellular calcium concentrations and activation of proteolytic enzymes. These enzymes destroy muscle cells and release large amounts of potassium, phosphate, myoglobin, aldolase, creatine phosphokinase (CPK), lactate dehydrogenase, aspartate transaminase, and urate into the bloodstream (Khan, 2009 ▶; Zager, 1996 ▶; Wang et al., 2011 ▶). Pain, muscle weakness, dark urine, fever, weakness and lethargy, tachycardia, nausea, and vomiting are clinical symptoms of RM; dark-colored urine is often due to the presence of myoglobin (Petejova and Martinek, 2014 ▶). 

AKI is a severe disorder that is caused by rhabdomyolysis in approximately 10 to 40% of cases. Different observations show that the main element in induction of AKI following RM, is myoglobin. This protein is freely filtered by the glomeruli and may cause tubular obstruction and damage. The mechanisms underlying myoglobin-induced kidney damage include vasoconstriction, obstruction of the distal tubule due to cast formation, and direct toxicity of myoglobin to renal tubular cells. Inside the tubular cell, myoglobin ferrous is oxidized and converted to ferric form, leading to the formation of hydroxyl radicals. Reactive oxygen species (ROS) generation in turn induces lipid peroxidation and the destruction of mitochondrial membranes occurs and causes the production of inflammatory cytokines such as interleukin 1β (IL-1β) and tumor necrosis factor-α (TNF-α) from endothelial and tubular cells, which leads to kidney tissue damage and dysfunction. The effect of ROS on endothelial cells also reduces NO availability and causes ischemia (Ragheb et al., 2009 ▶). During RM, the volume of circulating blood also decreases due to extracellular fluid leakage into the damaged tissue. Activation of the renin-angiotensin system by contracting renal arteries also reduces renal blood flow and then disrupts the small blood circulation to the kidneys, resulting in hypoxia (Petejova and Martinek, 2014 ▶; Baliga et al., 1999 ▶). Tubular casts are caused by the reaction of myoglobin and Tamm-Horsfall protein which produces brown casts. Various approaches are used in treating RM, the most important of which being the administration of diuretics and the use of antioxidants. The diuretic agents reduce fluid accumulation in the interstitial space and the accumulation of myoglobin in the kidney tissue and cast formation, thereby glomerular filtration rate (GFR) and renal blood flow are increased. 

Antioxidant agents also prevent lipid peroxidation and kidney tissue damage. *Nigella sativa* (NS) is an annual plant that belongs to Ranunculaceae family (Ghorbanli et al., 1999 ▶). NS contains non-volatile oils, volatile oils, alkaloids, saponins, and nutrients including protein, minerals, vitamins, and carbohydrates and it has traditionally been used to treat a wide range of diseases (Ragheb et al., 2009 ▶). Its pharmacological properties include antioxidant, analgesic, anti-inflammatory, and anti-cancer effects. The diuretic effect of NS has also been shown in various studies (Benhelima et al., 2016 ▶). Thymoquinone (2-isopropyl-5-methyl-1,4-benzoquinone) (TQ) is one of the active and primary chemical components of NS essential oil and the main therapeutic effects of NS are related to it (Ragheb et al., 2009 ▶). Due to the anti-inflammatory and antioxidant effects as well as the diuretic effect of NS and since no study has been done on the effect of NS on acute renal failure caused by RM (Ragheb et al., 2009 ▶; Yildiz et al., 2010 ▶; Hosseini et al., 2017 ▶), this study aimed to determine the effect of aqueous-alcoholic extract of NS and TQ on AKI induced by RM. 

## Materials and Methods

**Preparation of extract**

*Nigella sativa *seeds were obtained from the local market and identified by botanists in theherbarium of Ferdowsi University of Mashhad (herbarium number 293- 0303-1). In this study, first, 70% ethanolic extract of NS was prepared by the distillation method and using the Soxhlet apparatus. A rotary evaporator was used to concentrate the extract. It was then kept at 4^o^C until it was used.

**Chemicals**

TQ (Sigma-Aldrich Co., USA) was prepared in saline and dimethyl sulfoxide (DMSO). Trichloroacetic acid (TCA), 5, 5´-dithiobis-2-nitrobenzoic acid (DTNB), Tris, ethylenediaminetetraacetic acid (EDTA), 2-thiobarbituric acid (TBA), potassium chloride (KCl), and hydrochloric acid (HCl) were obtained from Merck Company (Germany). 

**Animals**

In this study, 40 male Wistar rats weighing 220-300 g were used. Animals with free access to water and food were kept at 22 2±2°C and in the light-dark cycle for 12:12 hr. The ethics committee of Mashhad University of Medical Sciences in Mashhad, Iran, approved the experiments (Ethical code: IR.MUMS.MEDICAL.REC.1399.208).

**Experimental design**

The animals were randomly divided into five groups of 8 rats. 

**Figure 1:**
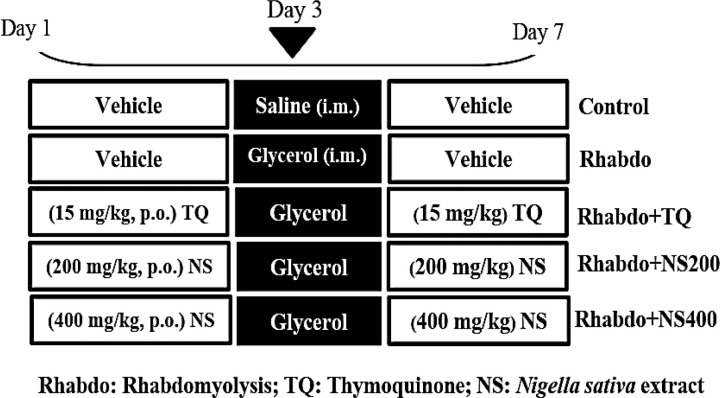
Groups and study design (n=8)

1) Control: In this group, the animals received TQ solvent orally for one week, and on the third day of the study, an intramuscular injection of normal saline equivalent to the volume injected for the glycerol, was performed. 

2) Rhabdomyolysis group (Rhabdo): In this group, the animals received TQ solvent orally for one week. On the third day of the study, 50% glycerol (10 ml/kg) was injected intramuscularly into the thigh muscle of both legs to induce RM (Singh et al., 2012 ▶). 

3) Rhabdo+TQ: Animals received TQ (15 mg/kg) by gavage for one week, and on the third day of the study, intramuscular injection of glycerol similar to the group 2 was performed (Hosseinian et al., 2017 ▶). 

4) Rhabdo+NS200: In this group, the animals received NS extract (200 mg/kg) by gavage for one week, and on the third day of the study, intramuscular injection of glycerol was performed similarly to group two. 

5) Rhabdo+NS400 group: In this group, the animals received NS extract (400 mg/kg) by gavage for one week, and on the third day of the study, intramuscular injection of glycerol was performed similar to the group 2 (Hosseinian et al., 2018 ▶). The grouping and study design are shown in Figure 1.

On day 0, the rats were weighed and placed in a metabolic cage. On day one, a 24-hr urine sample was collected, and blood samples were taken through the orbital sinus. Serum samples were also collected 24 hr after glycerol injection. At the end of the study (day 7), the animals were re-weighed and urine and serum samples were collected. Then, the kidneys were quickly removed and weighed and the animals were humanly killed. Serum levels of CPK, urea and creatinine were measured. Urine output as well as, urea and creatinine clearance (GFR) were calculated. For histological assessment, the right kidney was preserved at 10% formalin (hematoxylin-eosin staining), and the left kidney was kept at -80°C for oxidative damage assessments.

**Examination of histopathology**

After being fixed in 10% formalin, the right kidneys were dehydrated in graded alcohols and molded in paraffin. Then, 5-µm tissue slices were formed, and the slides were stained with the hematoxylin-eosin method to be used in microscopic examinations. Samples were scored between 0 and 4 as follows: 0= no tissue damage, 1=1-25% tissue destruction, 2=25-50% tissue destruction, 3=50-75% tissue destruction, and 4=75-100% tissue destruction. 

**Assessment of oxidative stress markers and antioxidant enzyme**

MDA, an oxidative stress index, forms a red-colored complex when it interacts with TBA, as a TBARS. It achieves its highest absorbance at 535 nm. In this part, 15 g TCA, 0.375 g TBA, and 2 ml HCl were combined, and 2 ml of this combination was mixed with 1 ml of serum or kidney homogenate in a centrifuge tube, which was then warmed for 50 min in a water bath. The mixture was centrifuged for 10 min at 1000 rpm after cooling. At 535 nm, the absorbance (A) of the colorful layer was measured (Uchiyama and Mihara, 1978 ▶). The following equation was used to calculate MDA concentration: 

C (M) = A/1.56×10^5^.

The renal tissue's total thiol content was calculated with the equation given by Sedlak and Lindsay (Sedlak and  Lindsay, 1968). 50 μl of supernatant was mixed with 1 ml of Tris-EDTA buffer. At 412 nm, absorbance was measured against Tris-EDTA buffer alone (A1). After that, 20 μl of the DTNB reagent (10 mM in methanol) was added to the mixture, and the sample absorbance was measured again after 10 min (A2). As a blank, the absorbance of DTNB reagent was measured (B). The following equation was used to calculate the total thiol concentration (mM):

Total thiol concentration (mM) = (A2-A1-B) × 1.07/0.05 × 13.6

Catalase activity in kidney tissue homogenates was determined using Aebi's calorimetric technique, which relies on the disappearance of hydrogen peroxide (H_2_O_2_) (Aebi, 1984 ▶). SOD activity was measured by the method described by Madesh and Balasubramanian (Madesh and Balasubramanian, 1998 ▶).

**Statistical analysis**

The data is reported as means±SEM. The difference in means of parametric data among groups was statistically analyzed using one-way analysis of variance (ANOVA) and the Tukey *post hoc* test. When the p-value was less than 0.05, the differences were considered statistically significant.

## Results

**Serum biochemical parameters and kidney function tests**

On the first day of the study, there was no significant change in serum levels of CPK, urea, creatinine, GFR, urea clearance or urine output among the different groups. Twenty-four hours after glycerol injection (day 4), serum levels of CPK, urea, creatinine and urine output showed a significant increase and urea clearance and GFR showed a significant decrease when compared to the control group (p<0.001 for all). On day 4, treatment of rats with NS extract and TQ significantly decreased serum levels of CPK, urea, creatinine and urine output when compared to the Rhabdo group (p<0.05- p<0.001). In these groups, urea clearance showed significant increase compared to the Rhabdo group (p<0.05-0.001). GFR significantly increased in Rhabdo+NS 200 and 400 groups when compared to the Rhabdo group (p<0.05 for both). On day 4 in the Rhabdo+NS200 and Rhabdo+NS400 groups, serum urea and creatinine levels showed significant decrease as compared to the Rhabdo+TQ group (p<0.05-p<0.001). Interestingly, there was no significant change in these parameters between NS (200 and 400 mg/kg) treated and control groups.

**Figure 2:**
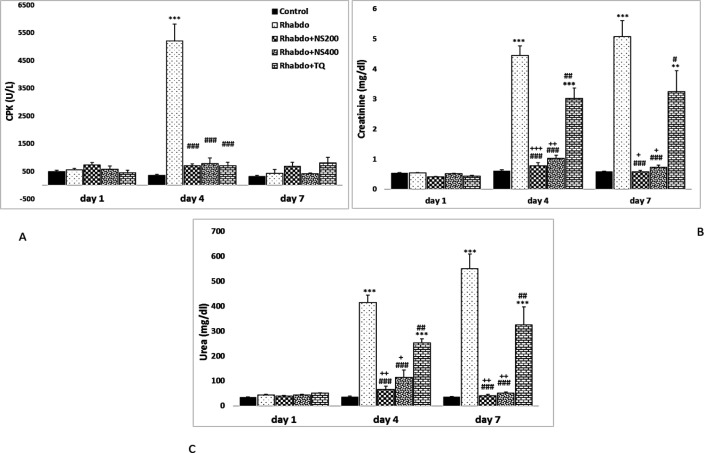
Serum levels of creatine phosphokinase (CPK) (A), creatinine (B), and urea (C) in the experimental groups. The values represent mean±SEM. Rhabdo: rhabdomyolysis; NS200: *N*igella *sativa (*200 mg/kg), NS400: *Nigella sativa* (400 mg/kg), and TQ: thymoquinone. *p<0.05, **p<0.01, and ***p<0.001 compared to the control group. ^#^p<0.05, ^##^p<0.01, and ^###^p<0.001 compared to the Rhabdo group.^ +^p<0.05, ^++^p<0.01, and ^+++^p<0.001 compared to the TQ group

**Figure 3:**
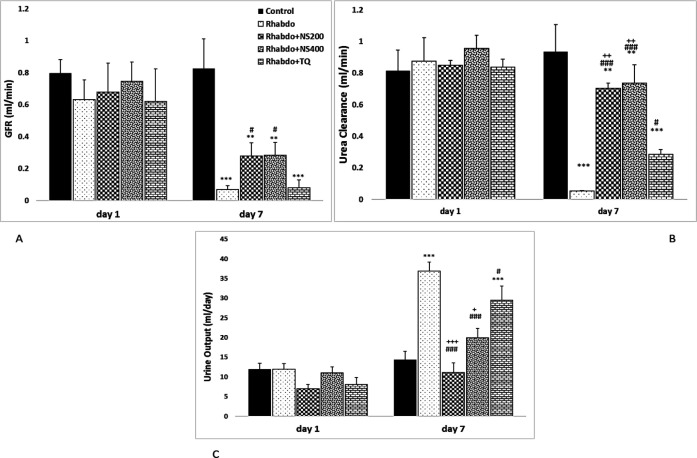
Glomerular filtration rate (GFR) (A), urea clearance (B), and urine output (C) in the experimental groups. The values represent mean±SEM. GFR: Glomerular Filtration Rate; Rhabdo: rhabdomyolysis; NS200: *N*igella *sativa (*200 mg/kg), NS400: *Nigella sativa* (400 mg/kg), and TQ: thymoquinone. ***p<0.001 compared to control group. ^#^p<0.05, and ^###^p<0.001 compared to the Rhabdo group.^ +^p<0.05, ^++^p<0.01, and ^+++^p<0.001 compared to the TQ group

On the last day of the study (day 7), serum level of CPK showed no significant change among the different groups. However, on the 7^th^ day, serum urea and creatinine, and urine output were significantly higher in the Rhabdo group when compared to the control group (p<0.001 for all) (Figures 2 & 3). On day 7 in the Rhabdo group, GFR and urea clearance significantly decreased compared to those of the control group (p<0.05- p<0.001) (Figure 2). In this day, in the groups treated with NS extract and TQ, there was no significant change in serum CPK when compared to the Rhabdo group. However, NS extract and TQ significantly decreased serum levels of urea and creatinine when compared to the Rhabdo group (p<0.05- p<0.001). Urine output in the Rhabdo+NS200, Rhabdo+NS400, and Rhabdo+TQ groups decreased significantly on the 7th day when compared to the Rhabdo group (p<0.05-0.001). Additionally, the Rhabdo+NS200 and Rhabdo+NS2400 groups had significantly lower urine output than the Rhabdo+TQ group (p<0.05-0.001). Urea clearance and GFR in the NS (200 and 400 mg/kg)- and TQ-treated groups significantly increased when compared to the Rhabdo group (p<0.05-0.001). These parameters, were significantly higher in the Rhabdo+NS 200 and 400 mg/kg groups compared to the TQ-treated animals (p<0.05-0.001) (Figure 3).

**Oxidant/antioxidant balance in the kidney**

**Figure 4:**
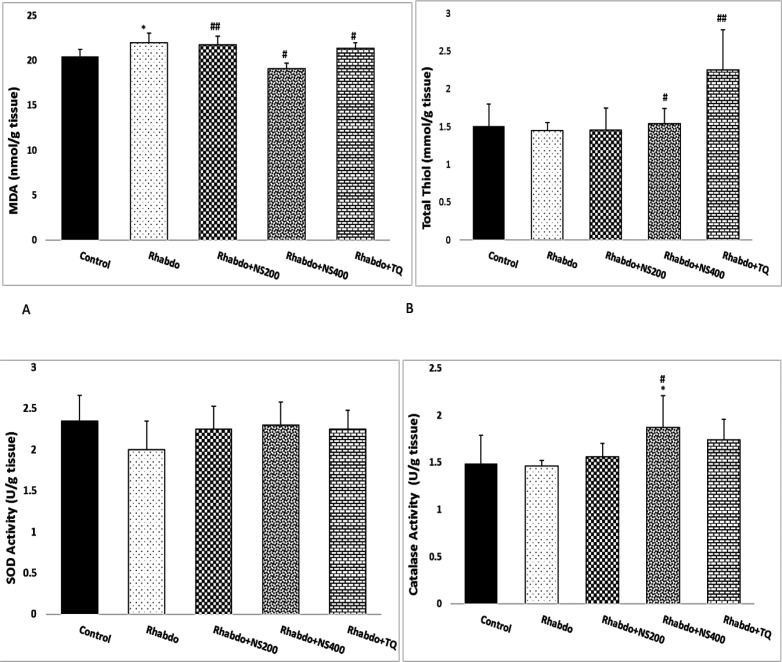
Renal tissue concentrations of malondialdehyde (MDA) (A), total thiol content (B), superoxide dismutase (SOD) activity (C) and catalase activity (D) in all experiment groups. The values represent mean±SEM. MDA: malondialdehyde; SOD: superoxide dismutase; Rhabdo: rhabdomyolysis; NS200 *Nigella sativa* (200 mg/kg)), NS400 *Nigella sativa* (400 mg/kg), and TQ (thymoquinone). *p<0.05 compared to the control group. *p<0.05 compared to the control group, and ^#^p<0.05 compared to the Rhabdo group

 Renal MDA concentration in the Rhabdo group significantly increased when compared to the control animals (p<0.05). 

in the Rhabdo+NS200, Rhabdo+NS400, and Rhabdo+TQ groups kidney tissue level of MDA significantly decreased when compared to the Rhabdo group (p<0.05-0.01). MDA level showed no significant alteration between the TQ- and NS-treated rats (Figure 4A). 

Total thiol content in kidney tissues of the Rhabdo group showed no significant change when compared to the control group. Administration of the NS extract and TQ in the Rhabdo+NS400 and Rhabdo+TQ groups significantly increased total thiol content when compared to the Rhabdo group (p<0.05-0.01) (Figure 4B).

SOD and catalase activities were not significantly different in the Rhabdo group compared with the control animals. SOD activity in NS extract and TQ treated groups showed no significant alteration when compared to Rhabdo group (Figure 4C).

However, catalase activity in the Rhabdo+NS400 group was significantly higher than those of Rhabdo animals (p<0.05) (Figure 4D). 

**The effect of NS extract and TQ on kidney pathological changes**

**Figure 5:**
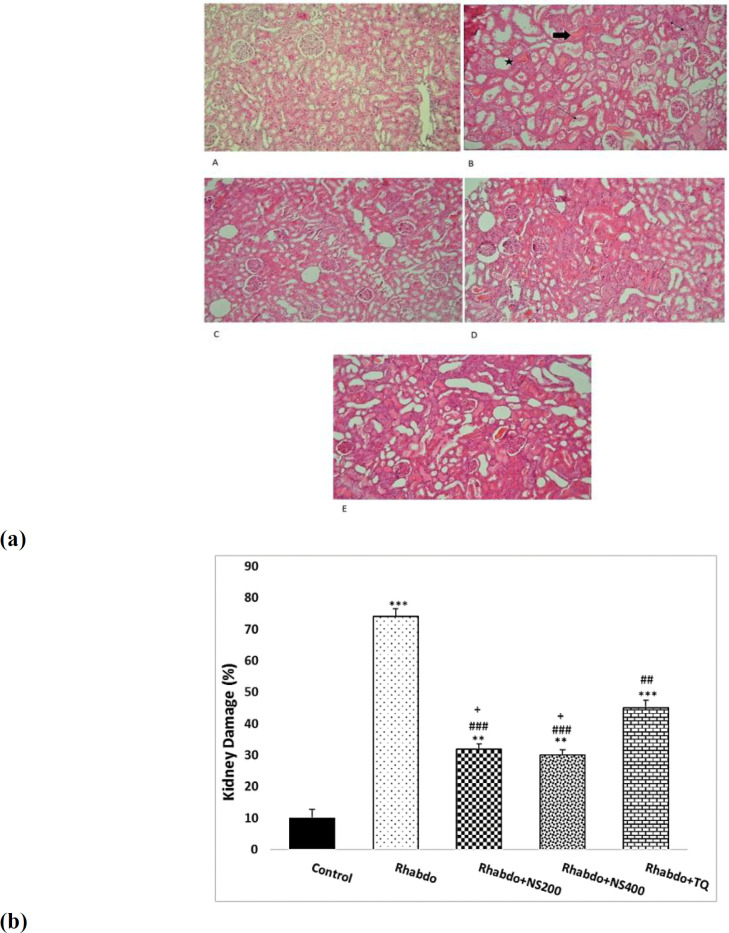
(a) Light microscopy of renal sections in day 7 in experimental groups. The morphology of the kidneys in controls animals was normal. Tubular dilatation (star), hyaline casts (thick arrow), tubular necrosis (thin arrow) and interstitial infiltration (double arrow) were all observed in the Rhabdo group. Treatment with the NS extract and TQ caused mild tubular, glomerular, and interstitial changes in the Rhabdo rats (hematoxylin and eosin, 400). (b) The values represent mean±SEM. Rhabdo: rhabdomyolysis; NS200: *Nigella sativa* (200 mg/kg), NS400: *Nigella sativa* (400 mg/kg), and TQ (thymoquinone). **p<0.01 and ***p<0.001 compared to the control group. ^##^p<0.01 and ^###^p<0.001 compared to the Rhabdo group. ^+^p<0.05 compared to the TQ group

Kidney tissue sections in the control group revealed normal architecture when compared to Rhabdo group (Figure 5A). Desquamation, necrosis, and vacuolation in epithelial cells, hyaline casts and necrotic epithelial cells in tubule lumens, and hemorrhage were the tubular and glomerular degenerative changes caused by RM (Figure 5B). Compared to the Rhabdo group, treatment with the NS extract and TQ improved kidney histological abnormalities (Figure 5C, D, and E). The Rhabdo group had significantly higher kidney damage than the control group (p<0.001). Treatment with the NS extract and TQ significantly improved kidney histopathology characteristics as compared to the Rhabdo group (p<0.01- p<0.001). Kidney damage in the Rhabdo+NS200 and Rhabdo+NS400 groups was significantly lower when compared to Rhabdo+TQ group (p<0.05) (Figure 5b).

## Discussion

Rhabdomyolysis (RM) is a life-threatening disease that arises as a consequence of skeletal muscle injury and can result in acute tubular damage to the kidneys due to the release of myoglobin by injured myocytes (Panizo et al., 2015 ▶). Vasoconstriction and ischemia, nephrotoxicity and tubular obstruction by myoglobin casts are the mechanisms by which, RM causes renal failure (Homsi et al., 1996 ▶). One of the most common laboratory models for inducing AKI following RM is the intramuscular injection of glycerol after a period of 12 to 24 hr of water deprivation (Paller, 1988 ▶). In the present study, in order to induce RM, a single intramuscular injection of 50% glycerol was used on the third day of the study. The results showed that in the Rhabdo group, glycerol administration caused a significant increase in CPK enzyme as well as serum urea and creatinine concentrations 24 hr after glycerol injection (day four of the study). Also, in the Rhabdo group, on the last day of the study, urine output and urea and creatinine clearance showed significant changes compared to the control group. In the current study, the results of oxidant/antioxidant balance showed that glycerol administration induced lipid peroxidation and nearly 17% and 15% decrease in total thiol groups and the activity of SOD enzyme, respectively. The percentage of kidney tissue damage also increased in line with kidney disfunction. Our findings agreed with previous studies (Korrapati et al., 2012 ▶; Chander et al., 2003 ▶; Mousleh et al., 2018 ▶). Following rupture of the muscle cell membrane, extracellular fluid enters the muscle cell. This volume depletion and following decrease in renal blood flow, activates the renin-angiotensin system, resulting in constriction of the renal arteries and ultimately reducing GFR (Panizo et al., 2015 ▶). On the other hand, decreased renal blood flow reduces the rate of fluid flow inside the tubules and therefore the reaction of myoglobin with Tamm-Horsfall protein and the formation of casts inside the tubules that could in turn, reduce the GFR (Panizo et al., 2015 ▶). Therefore, increasing serum CPK, decreasing GFR and subsequent accumulation of nitrogenous wastes such as urea and creatinine, are the characteristics of induction of RM and AKI caused by it, which were well seen in the present study. Studies have shown that oxidative stress is the main molecular mechanism in the pathophysiology of AKI following RM. Following RM, large amounts of myoglobin are filtered through the glomeruli and endocytosed by tubular cells through megalin-cubilin receptors. Inside the tubular cell, the ferrous (Fe^2+^) form of myoglobin is converted to the ferric form (Fe^3+^) to form a hydroxyl radical (the most reactive ROS). These free radicals cause lipid peroxidation of membrane fatty acids, resulting in the production of MDA (Gburek et al., 2003 ▶). However, in the present study, in RM group the level of antioxidant factors such as total concentration of thiol groups and activity of SOD and catalase enzymes didn’t show a significant decrease when compared to control group. These findings may be due to the time-dependent activation of oxidative stress pathways in the kidney following RM, as in most studies oxidative stress has occurred between 24 and 48 hr after RM (Yavuz et al., 2018 ▶; Zhang et al., 2017 ▶; Aydogdu et al., 2006 ▶, Roa Konda et al., 2016 ▶). 

In this study, treatment of rats with RM with the NS extract and to some extent TQ, showed a considerable improvement in serum biochemical parameters and kidney function tests 4 days after glycerol injection. Interestingly, the NS extract in both doses used in this study was able to bring serum levels of urea and creatinine to those of the control group. Based on our findings, the NS extract and TQ could improve the lipid peroxidation in rats with RM. However, only the NS 400 mg/kg could increase the thiol content and the activity of catalase. The exact mechanisms of nephroprotective effects of the NS extract and TQ are not fully elucidated. Nonetheless, considering the main role of oxidative stress and inflammation in the pathophysiology of AKI caused by RM on the one hand, and the strong antioxidant and anti-inflammatory effects of black seed on the other hand, the positive results of the present study can be justified (Hosseinian et al., 2018 ▶; Hosseinian et al., 2017 ▶). Also, the direct inhibitory effect of NS on renin-angiotensin system, as well as, inhibition of the contractile effects of ROS on renal arteries can explain some of the beneficial effects seen in the present study (Hosseinian et al., 2018 ▶; Hosseinian et al., 2017 ▶). In addition, the reason for the better effect of NS extract than TQ in improving kidney damage due to RM, could be due to the fact that in addition to TQ, other compounds in the plant also play an effective role in protective and beneficial effects. 

The present study showed that total *Nigella sativa* extract and thymoquinone had a good protective effect on renal function in animals with rhabdomyolysis. It is possible that part of these beneficial effects of *N. sativa* extract and thymoquinone is due to their antioxidant and anti-inflammatory effect.

## Conflicts of interest

The authors have declared that there is no conflict of interest.
